# Open-source automated insulin delivery shows comparable glycemic outcomes to a commercial hybrid closed-loop system in real-world type 1 pediatric diabetes care: a retrospective cohort study from British Columbia, Canada

**DOI:** 10.48130/pedi-0026-0008

**Published:** 2026-09-08

**Authors:** Duha Hejla, Kate Hawke, Leah Borovoi, Tom Elliott, Constadina Panagiotopoulos

**Affiliations:** 1 Endocrine & Diabetes Unit, Department of Pediatrics, BC Children's Hospital, Vancouver, British Columbia V6H 3V4, Canada; 2 BC Diabetes, Vancouver, British Columbia, Canada; 3 Bar-Ilan University, Azrieli Faculty of Medicine, Safed, Israel; 4 BC Children's Hospital Research Institute, University of British Columbia, Vancouver, British Columbia V5Z 4H4, Canada

**Keywords:** Type 1 diabetes, Open-source automated insulin delivery, Hybrid closed-loop systems, Glycemic control, Insulin pump therapy

## Abstract

Open-source automated insulin delivery shows promise for managing type 1 diabetes, though pediatric efficacy and safety data remain limited. This study aimed to compare real-world glycemic outcomes of open-source automated insulin delivery systems with hybrid closed-loop systems in youth with type 1 diabetes. This retrospective cohort study included individuals aged ≤ 19 years with type 1 diabetes for at least one year who used either open-source automated insulin delivery systems (Loop, iAPS, or AndroidAPS) or hybrid closed-loop systems (Tandem t:slim) for at least six months between January 2015 and June 2024. Data were collected from one month before system initiation to 18 months after initiation or the last clinical visit. Glycated hemoglobin, time in range, and glucose management indicator were evaluated using regression analyses and adjusted linear mixed-effects models. A total of 198 participants (143 open-source automated insulin delivery and 55 hybrid closed-loop users) were included (mean age 13.8 years; 57% male). Over 18 months, glycated hemoglobin decreased from 7.40% to 6.96% in the open-source automated insulin delivery group and from 7.35% to 7.28% in the hybrid closed-loop group. Adjusted analyses showed no significant differences in glucose management indicator or time in range between groups across follow-up. Severe hypoglycemia occurred in one participant per group, and no diabetic ketoacidosis events were observed. Open-source automated insulin delivery systems demonstrated similar long-term glycemic outcomes and safety compared with hybrid closed-loop systems, supporting their use as a safe, non-inferior alternative.

## Introduction

Automated insulin delivery (AID) systems—also referred to as hybrid closed-loop (HCL) or artificial pancreas systems—integrate continuous glucose monitoring (CGM), insulin pumps, and algorithm-driven insulin dosing. These technologies have revolutionized type 1 diabetes (T1D) management by improving glycemic control, reducing hypoglycemia, and alleviating the daily burden of diabetes care, as demonstrated in randomized controlled trials and real-world studies across both pediatric and adult populations^[1−4]^. Despite these benefits and regulatory approvals in many high-income countries, adoption of commercial AID systems remains inconsistent due to factors such as high cost, proprietary hardware and software limitations, regulatory hurdles, and prolonged innovation cycles^[5−7]^.

In response to these barriers, the diabetes community has pioneered open-source AID (OSAID) systems, including OpenAPS, AndroidAPS, Loop, iAPS, and Trio. These do-it-yourself (DIY), user-led systems leverage commercially available CGMs and insulin pumps to create flexible, customizable solutions outside of the commercial product ecosystem. Since the release of OpenAPS in 2015, global adoption has steadily increased. By March 2024, at least 3,262 individuals had self-reported OSAID use, though the actual number is likely higher^[8−10]^.

An expanding body of evidence in adults from both observational studies and randomized trials demonstrates that OSAID systems can achieve glycemic outcomes comparable to commercial HCL systems, without increasing the risk of severe hypoglycemia or diabetic ketoacidosis (DKA)^[10−12]^. Reflecting this evidence, both the American Diabetes Association and the European Association for the Study of Diabetes now recommend that healthcare professionals support patients who choose to use these systems^[2,3]^. While recent international consensus statements offer preliminary clinical guidance, further research is needed to establish the role of OSAID systems in routine care and to assess their long-term outcomes across diverse populations^[13]^. Despite growing clinical use among children and adolescents, there is limited research evaluating OSAID safety and efficacy in pediatric populations in the real-world setting^[14]^.

This study aimed to compare glycemic outcomes and safety between youth with T1D using OSAID systems and those using the Tandem t:slim HCL system. Further, we sought to evaluate how socioeconomic status relates to glycemic outcomes across both user groups.

## Materials and methods

### Study design

The study was conducted at BC Children's Hospital and BC Diabetes in Vancouver, British Columbia, Canada, between June 2024 and December 2024. This was a retrospective cohort study that included children and adolescents aged 19 years or younger with T1D for at least one year, who had used either an OSAID system built and supported by BC Diabetes or the HCL system (Tandem t:slim) for a minimum of six months between January 2015 and June 2024.

Glycemic data were collected from Nightscout™, Dexcom Clarity®, and both electronic and paper medical records. Data were extracted from the time of system initiation through 18 months post-initiation, or until the last available clinic visit, whichever occurred first. Baseline A1C was defined separately as the closest available measurement obtained within the 12 months preceding system initiation. Outcomes were assessed at approximately three-month intervals (three, six, nine, 12, and 18 months) and included hemoglobin A1C (A1C), percent time in range (TIR; 70–180 mg/dL [3.9–10.0 mmol/L]), and glucose management indicator (GMI). All longitudinal analyses were based on observed clinical and CGM data available at each scheduled follow-up time point. Consequently, the number of participants contributing data varied across time points because not all participants had data available at every follow-up visit. For A1C, the laboratory value closest to each target time point within ± three months was used.

In addition to glycemic outcomes, sociodemographic and clinical variables were collected. This included age, sex, height, weight, duration of diabetes, duration of insulin pump therapy, and patient postal code at time of enrollment. Height and weight were used to calculate body mass index (BMI), which was subsequently standardized for age and sex to generate BMI z-scores (zBMI)^[15,16]^.

Safety was assessed by reviewing for the occurrence of DKA and severe hypoglycemia documented in the clinical and electronic health record. DKA was defined as hyperglycemia (blood glucose > 11 mmol/L [≈200 mg/dL]), evidence of metabolic acidosis (venous pH < 7.30 and/or bicarbonate < 18 mmol/L), and ketonemia (blood *β*-hydroxybutyrate ≥ 3 mmol/L) or moderate to large ketonuria. Severe hypoglycemia was defined as an event associated with altered mental status, seizures, or impaired physical functioning that required assistance from another person for recovery, including administration of glucagon. Potential DKA and severe hypoglycemia events were identified through systematic retrospective review of emergency department visits, hospitalizations, outpatient clinical documentation, and available laboratory data within the electronic health record. Candidate events were reviewed against the prespecified biochemical and clinical definitions by clinician investigators with expertise in pediatric diabetes. Event ascertainment was not blinded to treatment group because of the retrospective design. Ambiguous cases were resolved by consensus. Events were counted from pump initiation until the last available clinic visit while participants were using the assigned automated insulin delivery system; no post-discontinuation observation window was applied. Data were abstracted using a standardized data collection sheet; a formal central adjudication committee was not used.

### Socioeconomic status and deprivation index

To evaluate the relationship between socioeconomic deprivation and glycemic outcomes before and after insulin pump initiation, we used the British Columbia Index of Multiple Deprivation (BCIMD). BCIMD is a provincial-level, area-based composite framework derived from Canadian census data to measure socioeconomic disparities and marginalization. It represents a BC-specific adaptation of the national Canadian Index of Multiple Deprivation (CIMD), which is a validated area-level index developed by Statistics Canada and captures deprivation across four key dimensions: economic dependency, residential instability, ethnocultural composition, and situational vulnerability (Supplementary Table S1).

Deprivation scores are assigned at the level of dissemination areas—small geographic units typically containing 400 to 700 residents—using domain-specific indicators derived from census data^[17]^. These indicators are combined using factor analysis, and the resulting scores are ranked and categorized into quintiles, ranging from 1 (least deprived) to 5 (most deprived).

Six-digit residential postal codes were collected from participants' medical records at baseline and linked to BCIMD scores using the 2016 Census and the most recent postal code conversion files (2020) from Statistics Canada. This approach allowed us to assign each participant an area-level deprivation score, serving as a proxy for individual-level socioeconomic status.

### Statistical analysis

All analyses were performed in Python 3.9 using pandas and NumPy for data handling, Matplotlib and seaborn for visualization, SciPy for inferential tests, stats-models for mixed-effects models, and scikit-learn for exploratory random forest models. All longitudinal glycemic analyses used observed data available at each time point.

Continuous baseline variables and unadjusted between-group outcome differences were compared using Welch's t-tests, and categorical variables were compared using *χ*^2^ tests or Fisher's exact tests, as appropriate. Multicollinearity was assessed using variance inflation factor (VIF) analysis.

Multiple linear regression models were developed to examine predictors of glycemic outcomes. Pearson correlation analyses were performed to evaluate associations between baseline and post-pump glycemic measures. Random forest regression models were also trained to assess feature importance, with model performance evaluated using MAE, MSE, RMSE, and *R*^2^.

Observed mean glycemic outcomes were summarized at baseline and three, six, nine, 12, and 18 months. Unadjusted between-group differences at each time point were calculated as OSAID minus HCL with 95% confidence intervals from Welch's t-tests.

To account for repeated measures and potential confounding, we also fit adjusted linear mixed-effects models with a participant-specific random intercept. Models included fixed effects for time (categorical), group, and time × group interaction, and were adjusted for age, sex, diabetes duration, and pump therapy duration. Adjusted between-group differences (OSAID minus HCL) and 95% confidence intervals at each time point were derived from model contrasts.

Collinearity checks revealed that individual deprivation indices were highly correlated. To address this issue and prevent multicollinearity in the regression models, we replaced the separate deprivation indices with a single composite measure—Deprivation Average—calculated as the mean of the four BCIMD domain scores. This composite score provided a more stable and interpretable model while retaining the multidimensional nature of deprivation.

## Results

### Participants' demographics and baseline characteristics

A total of 198 participants were included, with 143 in the OSAID group and 55 in the HCL group. Baseline characteristics were generally similar between groups (Table 1, Supplementary Tables S2 and S3). The OSAID group had a slightly shorter duration of diabetes (5.6 vs 6.5 years; *p* = 0.175) and insulin pump therapy duration (0.9 vs 1.7 years; *p* < 0.001) compared with the HCL group. BMI z-scores were modestly lower in the OSAID group (*p* = 0.004), whereas baseline glycemic indices (A1C, GMI, and TIR) were comparable. Socioeconomic deprivation data were missing for 33% of OSAID participants and none of the HCL participants. Among those with available data, OSAID users were less frequently in the least deprived quintiles for residential instability (19% vs 31%) and ethno-cultural composition (31% vs 45%) compared with HCL users, while HCL users were more represented in the most deprived quintile for economic dependency (33% vs 9%). The majority of participants in both groups were distributed across the middle quintiles (2–4), with 75% vs 60% for residential instability, 82% vs 64% for economic dependency, 54% vs 51% for ethno-cultural composition, and 60% vs 47% for situational vulnerability in the OSAID vs HCL group, respectively.

**Table 1:** Baseline characteristics of the study participants.

Baseline characteristic	Total (*n* = 198)	OSAID Group‡ (*n* = 143)	HCL Group† (*n* = 55)	*p*-value
Age, yr, mean (SD)	13.8 (3.9)	13.5 (3.9)	14.4 (3.6)	0.120
Sex, no. (%)				1.000
Male	113 (57)	82 (57)	31 (56)	
Female	85 (43)	61 (43)	24 (44)	
BMI *z* score*, mean (SD)	0.0 (1.0)	−0.2 (0.8)	0.4 (1.3)	0.004
British Columbia Index of Multiple Deprivation (BCIMD)¶, no. (%)				
Residential instability quintile				< 0.001
1 (least deprived)	35 (18)	18 (19)	17 (31)	
2	43 (22)	21 (22)	22 (40)	
3	37 (19)	34 (35)	3 (5)	
4	25 (13)	17 (18)	8 (15)	
5 (most deprived)	11 (6)	6 (6)	5 (9)	
Missing	47 (24)	47 (33)	0 (0)	
Economic dependency quintile				0.002
1 (least deprived)	10 (5)	8 (8)	2 (4)	
2	38 (19)	28 (29)	10 (18)	
3	41 (21)	31 (32)	10 (18)	
4	35 (18)	20 (21)	15 (27)	
5 (most deprived)	27 (14)	9 (9)	18 (33)	
Missing	47 (24)	47 (33)	0 (0)	
Ethno-cultural composition quintile				0.040
1 (least deprived)	55 (28)	30 (31)	25 (45)	
2	31 (16)	20 (21)	11 (20)	
3	27 (14)	21 (22)	6 (11)	
4	22 (11)	11 (11)	11 (20)	
5 (most deprived)	16 (8)	14 (15)	2 (4)	
Missing	47 (24)	47 (33)	0 (0)	
Situational vulnerability quintile				0.040
1 (least deprived)	60 (30)	34 (35)	26 (47)	
2	51 (26)	38 (40)	13 (24)	
3	22 (11)	12 (13)	10 (18)	
4	10 (5)	7 (7)	3 (5)	
5 (most deprived)	8 (4)	5 (5)	3 (5)	
Missing	47 (24)	47 (33)	0 (0)	
Duration of diabetes, yr, mean (SD)	5.9 (3.7)	5.6 (3.5)	6.5 (4.1)	0.175
Duration of pump therapy, yr, mean (SD)	1.1 (1.1)	0.9 (0.9)	1.7 (1.4)	< 0.001
Baseline A1C, %, mean (SD)	7.4 (1.1)	7.4 (1.2)	7.4 (1.0)	0.818
Baseline A1C, mmol/mol, mean (SD)	57.2 (12.4)	57.3 (12.9)	56.9 (11.0)	0.818
GMI#, %, mean (SD)	7.4 (0.9)	7.5 (1.0)	7.4 (0.6)	0.794
TIR***, %, mean (SD)	60.6 (14.5)	61.4 (14.0)	58.6 (15.6)	0.273
† HCL (hybrid closed-loop) group refers to participants using the Tandem t:slim X2 insulin pump platform during the study period. ‡ OSAID group refers to participants using open-source automated insulin delivery systems. * Body mass index (BMI) is expressed as a standard deviation (z) score relative to age- and sex-specific population norms. ¶ British Columbia Index of Multiple Deprivation (BCIMD) includes four components: residential instability, economic dependency, ethno-cultural composition, and situational vulnerability. Higher scores indicate greater deprivation. # The glucose management indicator (GMI) is an estimated A1C derived from continuous glucose monitoring (CGM) data. ** *p*-values compare OSAID versus HCL using Welch's t-test for continuous variables and *χ*^2^/Fisher's exact tests for categorical variables. Selected baseline measures use available-case denominators shown in Supplementary Tables S2 and S3. *** Time in range (TIR) refers to the percentage of time that glucose levels are within the target range of 3.9 to 10.0 mmol/L				

### Glycemic outcomes

Changes in glycemic outcomes over the 18-month follow-up are summarized in Table 2 and visualized in Fig. 1a–c. At baseline, A1C was 7.4% in OSAID and 7.4% in HCL using the prespecified laboratory-window definition. Unadjusted between-group A1C differences were small at three, six, nine, and 18 months, with the largest difference favoring OSAID at 12 months (−0.50%; 95% CI −0.92 to −0.08; *p* = 0.021), but not at 18 months (−0.33%; 95% CI −0.95 to 0.30; *p* = 0.292). GMI and TIR differences were not statistically significant at any follow-up time point. Adjusted mixed-effects models using the same observed outcome dataset and adjusting for age, sex, diabetes duration, and pump therapy duration yielded a similar interpretation: the estimated A1C difference (OSAID minus HCL) was −0.51% at 12 months (95% CI −0.90 to −0.12; *p* = 0.010) and −0.34% at 18 months (95% CI −0.81 to 0.12; *p* = 0.150). Adjusted differences in GMI and TIR remained small and non-significant across follow-up (Supplementary Table S4).

**Table 2:** Glycemic outcomes over 18 months in the open-source AID and HCL groups.

Metric	Time point (months)	N (OSAID)	OSAID mean (SD)	N (HCL)	HCL mean (SD)	Mean difference (OSAID – HCL, 95% CI)	*p*-value
A1C (%)	Baseline	120	7.4 (1.2)	46	7.4 (1.0)	–	–
	3	64	7.0 (0.8)	39	7.2 (0.9)	−0.25 (−0.59, 0.09)	0.148
	6	63	7.1 (0.8)	35	7.3 (0.9)	−0.23 (−0.58, 0.12)	0.200
	9	48	7.1 (0.9)	33	7.2 (0.7)	−0.04 (−0.38, 0.30)	0.803
	12	29	6.8 (0.7)	28	7.3 (0.9)	−0.50 (−0.92, -0.08)	0.021
	18	14	7.0 (0.9)	23	7.3 (0.9)	−0.33 (−0.95, 0.30)	0.292
GMI (%)	Baseline	134	7.5 (1.0)	49	7.4 (0.6)	–	–
	3	131	7.2 (0.6)	49	7.2 (0.5)	0.04 (−0.15, 0.22)	0.704
	6	110	7.3 (0.9)	48	7.2 (0.5)	0.10 (−0.12, 0.32)	0.364
	9	78	7.3 (0.9)	45	7.2 (0.5)	0.10 (−0.15, 0.35)	0.422
	12	40	7.4 (0.8)	43	7.3 (0.6)	0.12 (−0.20, 0.43)	0.465
	18	30	7.4 (0.8)	33	7.4 (0.5)	0.08 (−0.26, 0.41)	0.651
TIR (%)	Baseline	134	61.4 (14.0)	50	58.6 (15.6)	–	–
	3	131	63.7 (12.1)	49	65.7 (13.7)	−1.98 (−6.42, 2.46)	0.377
	6	110	64.3 (12.2)	48	64.7 (11.5)	−0.39 (−4.42, 3.65)	0.849
	9	78	63.9 (11.8)	45	63.8 (11.8)	0.11 (−4.26, 4.49)	0.959
	12	40	63.1 (12.1)	43	62.0 (12.7)	1.10 (−4.30, 6.51)	0.686
	18	30	62.1 (11.6)	33	61.9 (11.9)	0.19 (−5.73, 6.11)	0.949
OSAID = Open-Source Automated Insulin Delivery; HCL = Hybrid Closed-Loop; A1C = Hemoglobin A1C; GMI = Glucose Management Indicator; TIR = Time in Range; CI = Confidence Interval. Data represent observed mean (± SD) values for A1C (%), GMI (%), and TIR (%; glucose 3.9–10.0 mmol/L or 70–180 mg/dL) at each follow-up time point. The mean difference shown at each follow-up time point is OSAID minus HCL with its 95% CI based on Welch's t-test. Negative differences favor OSAID for A1C and GMI, whereas positive differences favor OSAID for TIR. All longitudinal outcome tables use the same observed outcome dataset. N reflects participants with available observed data for the corresponding outcome and time point. N varies due to availability of laboratory or CGM data at each time point.							

**Figure 1:**
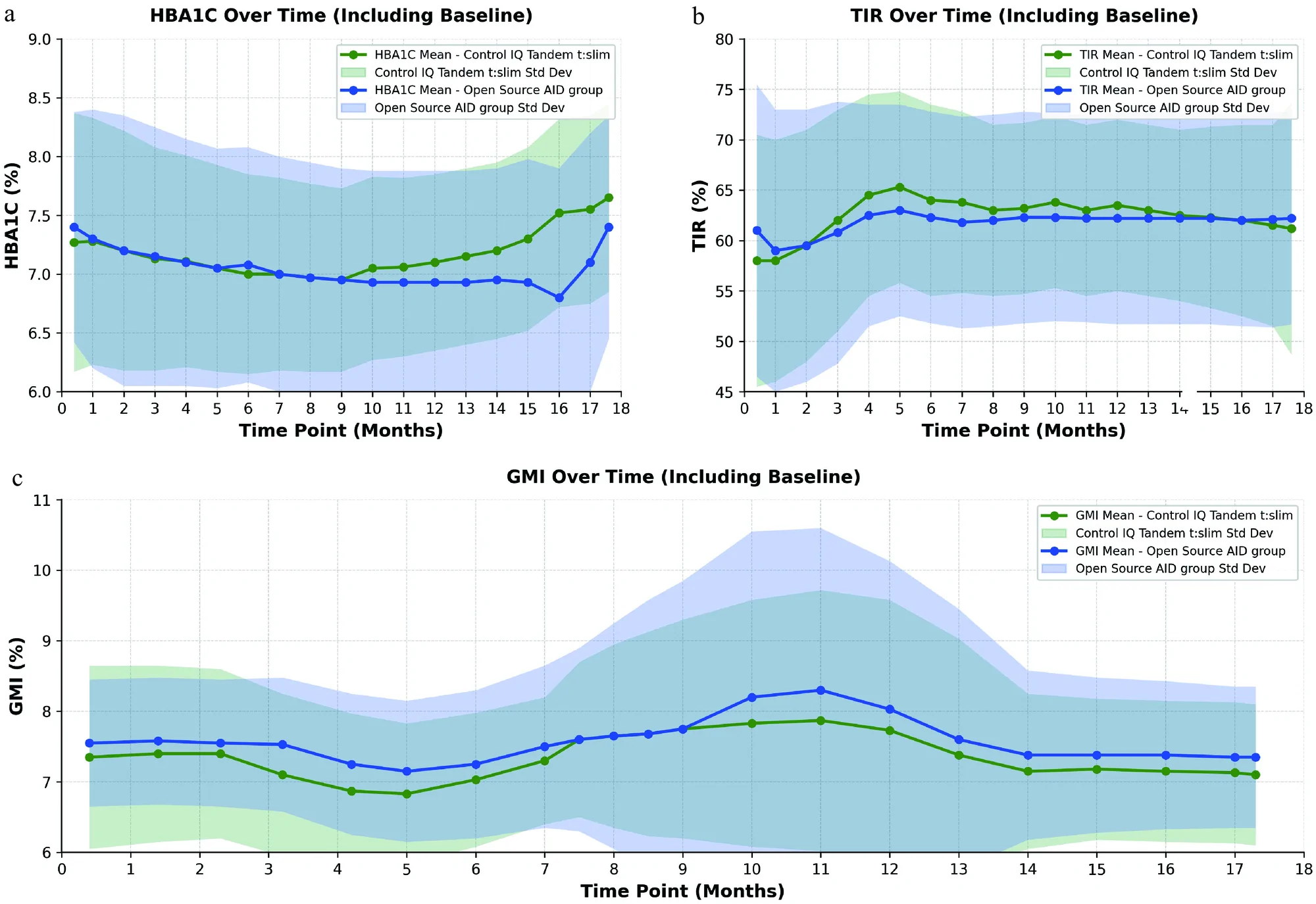
A1C, TIR, and GMI levels over time after initiation of insulin pump therapy. (a) Shown are the mean A1C values for participants using either the HCL system (green line) or the OSAID system (blue line), from baseline through 18 months after initiation of pump therapy. Values were derived from laboratory testing at scheduled follow-up visits. Shaded areas represent the standard deviation at each time point. (b) Shows the percentage of time that participants in the two study groups were within the target glucose range (time in range [TIR], defined as 3.9 to 10.0 mmol/L [70–180 mg/dL]). (c) Displays the glucose management indicator (GMI, expressed as a percentage), tracked from baseline through 18 months after the initiation of insulin pump therapy. Data are derived from continuous glucose monitoring. Lines represent group means, and shaded areas indicate standard deviations. The green line represents the hybrid closed-loop (HCL) group, and the blue line represents the open-source automated insulin delivery (OSAID) group.

### Socioeconomic deprivation and glycemic control before and after pump initiation

Multiple linear regression models were adjusted for age, diabetes duration, sex, and geographical location. These models showed no statistically significant association between deprivation and pre-pump glycemic outcomes, including A1C (*β* = 0.01, *p* = 0.84; adjusted *R*^2^ = 0.04), TIR (*β* = –0.33, *p* = 0.07; adjusted *R*^2^ = 0.10), or GMI (*β* = 0.01, *p* = 0.73; adjusted *R*^2^ = 0.09). Similarly, post-pump glycemic outcomes showed no significant independent relationship with deprivation once baseline control was included in the model (e.g., post-pump A1C: *β* = 0.03, *p* = 0.29; adjusted *R*^2^ = 0.59). However, when using random forest models, deprivation emerged as a moderately important predictor, contributing 12% to 17% of overall variable importance—less than age (33%–37%) and diabetes duration (21%–22%) but still non-negligible. The discrepancy between random forest and linear regression analyses regarding socioeconomic variables likely arises from random forests capturing complex, nonlinear interactions and subtle variable importance, whereas linear models only identify direct linear associations. Notably, Pearson correlation analyses confirmed that baseline glycemic status was the strongest determinant of post-pump outcomes, with strong linear correlations observed between pre- and post-pump values (A1C: r = 0.72; TIR: r = 0.61; GMI: r = 0.74; all *p* < 0.001).

### Safety outcomes

Safety analyses included all 198 participants: 143 in the OSAID group and 55 in the HCL group. Severe hypoglycemia occurred in 1/143 OSAID participants and 1/55 HCL participants (two total events); both participants experienced loss of consciousness accompanied by documented hypoglycemia. No DKA events were observed in either group (0/143 OSAID; 0/55 HCL).

## Discussion

In this 18-month, real-world, retrospective observational study—the first of its kind in Canadian pediatric patients—we show that glycemic outcomes in youth using OSAID systems are non-inferior to those using a commercial HCL system.

Specifically, both groups achieved reductions in A1C during the initial months following pump initiation, with the OSAID group demonstrating a slightly greater early decline. However, these improvements attenuated over time, and by 18 months, there were no statistically significant differences between the groups in A1C, GMI, and TIR at any follow-up point. This pattern was observed in both groups and was not unique to OSAID users. Although the OSAID group demonstrated a statistically significant lower A1C compared with HCL users at 12 months, this difference was no longer significant at 18 months, while both groups maintained overall improvements compared with baseline. Several factors may explain this trajectory. In real-world settings, early improvements following AID initiation may reflect increased clinical engagement, structured education, and intensive adaptation to a new technology, followed by stabilization as users and healthcare teams transition to routine long-term management. Furthermore, sustained glycemic outcomes may be influenced by factors not captured in this retrospective analysis, including adherence to device use, behavioral and lifestyle factors, insulin optimization, and differences in algorithm utilization. The smaller number of participants contributing data at later follow-up time points may have also reduced the ability to detect smaller between-group differences. Overall, these findings suggest that while both systems support improved glycemic control, neither demonstrated sustained superiority in this real-world setting. These findings align with prior real-world studies. Wu et al. reported that, in adults with T1D, OSAID systems were non-inferior to HCL systems in key glycemic outcomes, including TIR and A1C, with similar safety profiles and no significant differences in patient-reported outcomes^[18]^. Similarly, Landau et al. (AWeSoMe Study Group) directly compared glycemic outcomes for 26 pediatric OSAID users and 20 MiniMed 780G HCL users and found similar reductions in A1C and TIR at follow-up, with only minor differences in hypoglycemia exposure^[19]^.

Although overall glycemic outcomes were similar between groups at 18 months, we observed subtle differences in specific metrics over time. Improvements in TIR were slightly more pronounced at certain time points in the HCL group, whereas the OSAID group consistently maintained lower A1C levels. These patterns may reflect differences in system algorithms, user behavior, or adaptation periods. Supporting these observations, Landau et al. reported that pediatric OSAID and MiniMed 780G HCL users achieved comparable improvements in TIR and A1C, with the OSAID group maintaining a lower median A1C at baseline and follow-up despite similar TIR outcomes^[19]^. Similarly, real-world evidence indicates that while advanced AID systems improve both TIR and A1C, system-specific features may shape distinct glycemic profiles. For instance, OSAID users may attain lower A1C yet experience marginally increased time in hypoglycemia, reflecting differences in algorithmic TIR regulation^[20]^. Notably, in our cohort, these glycemic patterns were observed without excess adverse events, highlighting the reassuring safety of both systems across the 18-month follow-up. Each group experienced only one episode of severe hypoglycemia, and no cases of diabetic ketoacidosis were reported.

Our study found no significant association between socioeconomic deprivation and glycemic control—measured by TIR, GMI, and A1C—before or after insulin pump initiation using adjusted regression models; however, machine learning analyses suggested that deprivation may still modestly influence outcomes. In contrast, studies from Australia and the United Kingdom found that insulin pump therapy was associated with improved A1C across all socioeconomic strata, suggesting that pump benefits may be preserved regardless of deprivation status^[21,22]^. However, population-based data from Ontario revealed that adults with greater social disadvantage had worse glycemic outcomes and higher acute care use even after pump initiation, suggesting that technology alone may not eliminate disparities^[23]^.

Importantly, in this study, we found that the strongest predictors of post-pump glycemic outcomes were the corresponding baseline values, reinforcing the clinical reality that patients with poorer initial control often remain at risk for suboptimal outcomes despite access to advanced technologies. This finding aligns with previous studies demonstrating the persistence of glycemic trajectories over time^[24]^. This association may be attributable, in part, to early A1C levels serving as a surrogate marker for the extent of pancreatic beta cell destruction at disease onset, which in turn influences endogenous insulin secretory capacity and shapes the trajectory of future glycemic control^[24]^. In contrast, a more recent multi-center study from Australia has shown that higher baseline A1C can predict greater A1C reduction following initiation of AID systems in adults, with multivariable regression analyses consistently identifying baseline A1C as a key determinant of subsequent improvement, irrespective of system type or geographic region^[25]^. These emphasize the need for further longitudinal and mechanistic studies to better understand the interplay between initial glycemic status and long-term outcomes specifically in youth using advanced diabetes technologies.

### Strengths and limitations

This study should be interpreted within the context of its strengths and limitations. The relatively large sample size of this pediatric real-world study and the availability of socioeconomic deprivation index data enhance the robustness and generalizability of its findings compared to prior single-center or smaller cohort studies. The real-world nature of the data offers valuable insight into device performance and user experience. However, the limitations inherent to its retrospective design are acknowledged. Residual confounding is possible despite adjustment for key covariates (age, sex, diabetes duration, and pump therapy duration). As with any observational study, unmeasured factors such as patient adherence to insulin therapy, dietary habits, physical activity, diabetes education, family support, and other behavioral or clinical characteristics may have influenced glycemic outcomes and could not be fully accounted for. Therefore, causal inferences regarding comparative effectiveness should be interpreted with caution. Baseline C-peptide data (residual beta-cell function) were not routinely available and therefore could not be included in the analyses, which may influence glycemic trajectories. Longitudinal analyses relied on observed clinical and CGM data at each time point, and missing follow-up data—particularly laboratory A1C availability at later visits—may introduce selection bias. In addition, although the group-by-time interaction estimates differences in change from baseline between treatment groups, our primary analysis was based on a longitudinal linear mixed-effects model rather than a classical Difference-in-Differences design. Because only a single pre-intervention (baseline) measurement was available, the parallel pre-intervention trends assumption required for a classical Difference-in-Differences analysis could not be formally evaluated. The relatively short follow-up period may fail to capture longer-term outcomes or rare adverse events. Furthermore, the study population primarily consisted of well-controlled, 'tech-savvy' patients, which may limit the generalizability of the results to more diverse or underserved populations, and to health systems with limited access to insulin pumps, continuous glucose monitoring, or technical support for OSAID.

## Conclusions

This study demonstrated that both open-source AID and commercial HCL systems were associated with modest and comparable improvements in glycemic outcomes, with no sustained differences between groups in this pediatric population. Baseline A1C emerged as the strongest predictor of post-pump outcomes, and both systems demonstrated excellent safety profiles. Although adjusted regression analyses showed no significant association between socioeconomic deprivation and glycemic outcomes, exploratory machine learning suggested a modest potential influence. These findings highlight the importance of equitable access to AID technologies and support the role of OSAID as a viable option in settings where commercial systems remain inaccessible.

## SUPPLEMENTARY DATA

Supplementary data to this article can be found online.

## Data Availability

The data that support the findings of this study are available on request from the corresponding author. The data are not publicly available due to privacy or ethical restrictions.
