# Supplementary material for: Open-source automated insulin delivery shows comparable glycemic outcomes to a commercial hybrid closed-loop system in real-world type 1 pediatric diabetes care: a retrospective cohort study from British Columbia, Canada
Source: Pediatr Diabetes. 2026 Sep 8;2026:e009. doi: 10.48130/pedi-0026-0008 (PMC13599425; doi:10.48130/pedi-0026-0008)
Supplement: Supplementary file 1 [file pedi-2026-2026-1-0008-S1.zip › 10.48130_pedi-0026-0008-Suppl-TableS2.pdf]

Table S2. Deprivation Quintiles Distribution Among Both Groups

This table presents the distribution of sociodemographic characteristics across deprivation quintiles, from quintile 1 (least deprived) to quintile 5 (most deprived) among both groups. Data are reported as counts and percentages (n, %).

Deprivation Index	Group	Missing	Quintile 1 (N %)	Quintile 2 (N %)	Quintile 3 (N %)	Quintile 4 (N %)	Quintile 5 (N %)
Residential Instability	HCL	0 (0.00%)	17 (30.91%)	22 (40.00%)	3 (5.45%)	8 (14.55%)	5 (9.09%)
	OSAID	47 (32.87%)	18 (18.75%)	21 (21.88%)	34 (35.42%)	17 (17.71%)	6 (6.25%)
Economic Dependency	HCL	0 (0.00%)	2 (3.64%)	10 (18.18%)	10 (18.18%)	15 (27.27%)	18 (32.73%)
	OSAID	47 (32.87%)	8 (8.33%)	28 (29.17%)	31 (32.29%)	20 (20.83%)	9 (9.38%)
Ethno-cultural Composition	HCL	0 (0.00%)	25 (45.45%)	11 (20.00%)	6 (10.91%)	11 (20.00%)	2 (3.64%)
	OSAID	47 (32.87%)	30 (31.25%)	20 (20.83%)	21 (21.88%)	11 (11.46%)	14 (14.58%)
Situational Vulnerability	HCL	0 (0.00%)	26 (47.27%)	13 (23.64%)	10 (18.18%)	3 (5.45%)	3 (5.45%)
	OSAID	47 (32.87%)	34 (35.42%)	38 (39.58%)	12 (12.50%)	7 (7.29%)	5 (5.21%)
