# Supplementary material for: Open-source automated insulin delivery shows comparable glycemic outcomes to a commercial hybrid closed-loop system in real-world type 1 pediatric diabetes care: a retrospective cohort study from British Columbia, Canada
Source: Pediatr Diabetes. 2026 Sep 8;2026:e009. doi: 10.48130/pedi-0026-0008 (PMC13599425; doi:10.48130/pedi-0026-0008)
Supplement: Supplementary file 1 [file pedi-2026-2026-1-0008-S1.zip › 10.48130_pedi-0026-0008-Suppl-TableS3.pdf]

Table S3. Baseline Comparisons Between OSAID and HCL Groups (with p-values)

Baseline comparisons were performed using Welch's t-test for continuous variables and χ^2/Fisher's exact tests for categorical variables. Data are presented as mean (SD) for continuous variables and n (%) for categorical variables. Baseline glycemic variables use the same observed-data definitions as the main longitudinal analyses; Ns differ from the full cohort because availability varied by variable.

Variable	OSAID	HCL	P value
Age, years	n=143; 13.52 (3.92)	n=55; 14.45 (3.64)	0.120
BMI z-score	n=134; -0.18 (0.81)	n=54; 0.38 (1.28)	0.004
Diabetes duration, years	n=143; 5.66 (3.48)	n=55; 6.51 (4.08)	0.175
Pump therapy duration, years	n=143; 0.88 (0.89)	n=55; 1.73 (1.35)	<0.001
Baseline HbA1c, %	n=120; 7.40 (1.18)	n=46; 7.35 (1.01)	0.818
Baseline GMI, %	n=134; 7.45 (0.96)	n=49; 7.42 (0.65)	0.794
Baseline TIR, %	n=134; 61.37 (14.01)	n=50; 58.60 (15.56)	0.273
Male, n (%)	n=82; 82 (57.3%)	n=31; 31 (56.4%)	1.000
Female, n (%)	n=61; 61 (42.7%)	n=24; 24 (43.6%)	
