# Supplementary material for: Open-source automated insulin delivery shows comparable glycemic outcomes to a commercial hybrid closed-loop system in real-world type 1 pediatric diabetes care: a retrospective cohort study from British Columbia, Canada
Source: Pediatr Diabetes. 2026 Sep 8;2026:e009. doi: 10.48130/pedi-0026-0008 (PMC13599425; doi:10.48130/pedi-0026-0008)
Supplement: Supplementary file 1 [file pedi-2026-2026-1-0008-S1.zip › 10.48130_pedi-0026-0008-Suppl-TableS4.pdf]

Table S4. Adjusted Between-Group Differences from Linear Mixed-Effects Models

Linear mixed-effects models with participant-specific random intercepts included fixed effects for time (categorical), group, and time×group interaction, and were adjusted for age, sex, diabetes duration, and pump therapy duration. Values are adjusted differences (OSAID minus HCL) with 95% confidence intervals. N values indicate observed outcome data available at each time point in the same analytic dataset used for Table 2.

Outcome	Time (months)	N (OSAID)	N (HCL)	Adj. difference (OSAID–HCL), 95% CI	P value
A1C	0	120	46	0.065 (-0.245, 0.375)	0.680
A1C	3	64	39	-0.148 (-0.482, 0.186)	0.386
A1C	6	63	35	-0.194 (-0.535, 0.147)	0.265
A1C	9	48	33	-0.144 (-0.499, 0.211)	0.426
A1C	12	29	28	-0.512 (-0.903, -0.120)	0.010
A1C	18	14	23	-0.344 (-0.813, 0.124)	0.150
GMI	0	134	49	-0.073 (-0.331, 0.185)	0.580
GMI	3	131	49	-0.075 (-0.334, 0.183)	0.568
GMI	6	110	48	0.044 (-0.216, 0.304)	0.740
GMI	9	78	45	0.058 (-0.207, 0.324)	0.667
GMI	12	40	43	0.012 (-0.266, 0.289)	0.935
GMI	18	30	33	0.113 (-0.179, 0.405)	0.447
TIR	0	134	50	3.868 (-0.488, 8.223)	0.082
TIR	3	131	49	-0.770 (-5.134, 3.594)	0.729
TIR	6	110	48	-0.427 (-4.820, 3.967)	0.849
TIR	9	78	45	0.186 (-4.288, 4.659)	0.935
TIR	12	40	43	1.781 (-2.888, 6.449)	0.455
TIR	18	30	33	-1.036 (-5.931, 3.858)	0.678
