# Supplementary material for: Open-source automated insulin delivery shows comparable glycemic outcomes to a commercial hybrid closed-loop system in real-world type 1 pediatric diabetes care: a retrospective cohort study from British Columbia, Canada
Source: Pediatr Diabetes. 2026 Sep 8;2026:e009. doi: 10.48130/pedi-0026-0008 (PMC13599425; doi:10.48130/pedi-0026-0008)
Supplement: Supplementary file 1 [file pedi-2026-2026-1-0008-S1.zip › 10.48130_pedi-0026-0008-Suppl-TableS1.pdf]

Table S1. Description of Canadian Index of Multiple Deprivation (CIMD) Components – Statistics Canada

This table describes the Canadian Index of Multiple Deprivation, which comprises four dimensions reflecting different aspects of socioeconomic vulnerability.

Dimension	Description	Indicators
**Economic Dependency**	Reliance on non-employment income sources and reduced labor force participation	- Proportion aged 65+<br>- Proportion not in labor force (15+)<br>- Employment-to-population ratio<br>- Dependency ratio<br>- Proportion receiving government transfers
**Residential Instability**	Housing mobility and family structure characteristics	- Proportion of apartment dwellings<br>- Proportion of owner-occupied dwellings<br>- Proportion living alone<br>- Proportion who moved in past 5 years<br>- Proportion married/common-law
**Ethno-Cultural Composition**	Immigration, cultural diversity, and language isolation	- Proportion identifying as visible minorities<br>- Proportion of foreign-born individuals<br>- Proportion with no English or French knowledge<br>- Proportion of recent immigrants
**Situational Vulnerability**	Social and structural disadvantages	- Proportion identifying as Indigenous<br>- Proportion of dwellings needing major repair<br>- Proportion aged 25–64 without a high school diploma
